# Vicks’ Floral Guide

**Published:** 1887-01

**Authors:** 


VICK'S FLORAL GUIDE AND MONTHLY MAGAZINE for January has reached our table, and is a perfect boquet within itself, illustrated by hundreds of cuts of flowers of every description, and one full page colored chromo-lithograph of Pansies. It is also unusually full of interesting reading matter, which, without the flloral accessories, would make it a welcome visitor at any fireside. We welcome it to our exchange list.

